# The views of different care professionals on using an electronic health record in the care for older people

**DOI:** 10.1186/s12877-026-07977-8

**Published:** 2026-07-14

**Authors:** R. M. Vosters, Y. M.J. Goërtz, J. P.H. Hamers, K. Y.J. Sion

**Affiliations:** 1 Living Lab in Ageing and Long-Term Care, Maastricht University, Maastricht, The Netherlands; 2 Department of Health Services Research, Care and Public Health Research Institute, Faculty of Health, Medicine and Life Sciences, Maastricht University, Duboisdomein 30, Maastricht, 6229 GT The Netherlands; 3 MeanderGroep, Landgraaf, The Netherlands

**Keywords:** Electronic Health Record, Interprofessional collaboration, Nursing homes, Care for older adults

## Abstract

**Background:**

Interprofessional collaboration is proposed as an appropriate strategy to meet the increasingly complex care demands for older adults. The electronic health record (EHR) can support interprofessional teams in their daily work and quality improvement efforts. While various studies have reported on care professionals’ perspectives on the EHR, to date, no study has compared the views of different care professionals on EHR use. This study aimed to investigate how different disciplines of care professionals in nursing homes view the use of EHRs.

**Methods:**

A qualitative design was adopted. Six homogeneous focus groups were organized, with certified nurse assistants, registered nurses, elderly care physicians, and students in these three professions. Conventional content analysis was used to analyze the focus groups.

**Results:**

Forty-three (student) care professionals in the care for older adults participated in the focus groups. Analysis revealed three main themes. First, the EHR is mainly used as a communication tool, and further purposes, such as data-informed care, were pointed out as goals to be realized in the future. Second, better navigation of the EHR system necessitates skill development among EHR users and improvements in the technical functions of the EHR system. Finally, the EHR can support interprofessional collaboration in the care for older people. Yet, care professionals recognized that there is still room for improvement in such a collaboration.

**Conclusions:**

Different interpretations of what is considered good EHR reporting hindered effective collaboration. Proper collaboration with the EHR in the care for older adults requires increased digital literacy of care professionals, and EHR systems that are easier to use. Future studies should investigate what interprofessional teams need to better work together towards quality improvement in the care for older adults.

## Background

The aging population [1] presents challenges in the care for older adults. Nursing homes are experiencing time and resource constraints, high staff turnover [2], and an increasing demand for care [3]. Additionally, there has been a shift in the profiles of nursing home residents, exhibiting higher levels of disability and multimorbidity [4]. As a consequence, the delivered care is becoming increasingly complex [5]. This growing complexity necessitates close collaboration between various disciplines of professional caregivers and informal caregivers [6]. This so-called interprofessional approach is necessary as the demand for care exceeds the expertise of a single discipline [7]. While the terminology for the different disciplines differs among countries and settings [8], examples of disciplines in the care for older people are registered nurses, certified nurse assistants, physicians, physiotherapists, psychologists, and well-being professionals.

Interprofessional collaboration is defined as “an active and ongoing partnership often between people from diverse backgrounds with distinctive professional cultures […], who work together to solve problems or provide services” [9]. Interprofessional teams adopt an interdependent approach wherein team members are equally valued and are up-to-date on each other’s tasks and expertise [9]. They complement each other in their pursuit of a common goal [10].

Interprofessional collaboration has been shown to positively affect resident outcomes and support quality improvements, such as mobility enhancements and better care planning [11, 12]. In addition, another potential reason to adopt interprofessional collaboration is to facilitate and streamline care processes [10], which could reduce the burden on care professionals. Working in interprofessional teams thus holds promise for continuous quality improvement in the care for older people.

The electronic health record (EHR), also known as the electronic patient record or electronic medical record, is a tool that supports interprofessional teams in their work, as it is the central communication tool for care professionals to document and access information about each client [13–15]. The EHR contains a wealth of data that is recorded daily, such as medication intake, activities of daily living, fall incidents, and health status [16]. Studies on care professionals’ experiences working with EHRs in nursing homes have shown mixed results regarding their attitude towards working with the EHR. Benefits include access to more comprehensive information [17] and a decrease in human error in medication administration because of better documentation [14]. Moreover, care professionals perceive the quality of care they deliver to be higher following EHR implementation [18, 19], aligning with the predominantly positive health outcomes reported since EHR use [17]. However, there are also experienced challenges, including the structure of the EHR [15], limited IT-support, difficulties in transferring data to other care organizations [18], and higher costs [14].

Another challenge care professionals report is using EHR data for quality improvement purposes, despite indicating a wish to use EHR data for this purpose [20]. Several difficulties have been reported in this regard. First, EHRs contain a large amount of data that are not always well-structured [15]. Second, care professionals frequently lack sufficient support from their supervisors to effectively incorporate EHR quality data into their daily work [21]. And third, the reporting in EHRs is often inadequate [22], and incomplete [23], especially when it concerns residents’ personal information, such as beliefs, values, background information, and preferences [24]. To conclude, using EHR data for quality improvement is not straightforward, and care professionals need more support to use EHR quality data adequately.

Despite the abovementioned challenges, using EHR data for quality improvement presents opportunities. The EHR is a main method of communication in the care of older people. While previous research has highlighted care professionals’ experiences in using the EHR [15, 18], fewer studies compare the views of different disciplines of care professionals. Given the central role of the EHR in the daily interprofessional work of care professionals, understanding this topic from the various perspectives of care professionals is important.

Therefore, the objective of this study was to investigate how different disciplines of care professionals in Dutch nursing homes view the use of EHRs, and to identify similarities and differences between these views.

## Methods

### Study design

This study used a qualitative design, employing six focus groups to explore the experiences of (student) care professionals providing care for older people in nursing homes. Focus groups are particularly useful in understanding participants’ views and the reasons behind them. Additionally, this setting encourages interaction, allowing participants to clarify their opinions and build on each other’s views [25].

### Setting and participants

Long-term care in nursing homes in the Netherlands is divided into care for people with physical disabilities, called somatic care, and care for people with cognitive decline, such as dementia, known as psychogeriatric care [26]. The care staff consists of a range of professionals with different levels of education. Certified nurse assistants comprise most of the care staff in Dutch nursing homes, requiring two to three years of vocational education. A smaller proportion of the care in nursing homes is provided by registered nurses who hold a baccalaureate degree [27, 28]. In addition, the profession of elderly care physician is unique to Dutch nursing homes. Elderly care physicians are medically trained physicians with a specialization in the care of older people, and are employed by a nursing home organization [29]. This is in contrast to other countries where residents receive care from general practitioners who also have their own practice [30, 31].

Participants employed in a nursing home as elderly care physicians, registered nurses, certified nurse assistants, or students training for one of these roles were invited to take part in this study. A convenience sample of care professionals was recruited through the Living Lab in Ageing and Long-Term Care [32], the Dutch professional associations for elderly care physicians (Verenso), and the Dutch professional association for nurses and nurse aides (V&VN).

As this article, amongst others, aims to examine the similarities and differences in views between different disciplines of care professionals, the choice was made to organize homogeneous focus groups. As a result, common experiences within disciplines could be distilled from the focus groups. An additional benefit of this group composition is that it tends to foster a safe environment for sharing experiences between individuals with the same background [25]. Students pursuing a care profession degree were also included in separate focus groups, as they represent the future workforce and possess the latest knowledge on working with the EHR. They can therefore offer a unique perspective compared to professionals who have already been working with the EHR for a longer time. Both somatic and psychogeriatric care settings were represented in each focus group.

### Data-collection

Focus groups took place on site at the care professionals’ workplaces, at the university, or in the classroom during a scheduled education activity for the student care professionals. The focus group with elderly care physicians was conducted online, as this was preferred by the participants.

At the start of the first two focus groups, with certified nurse assistants and registered nurses, a photo elicitation technique was used. This method functioned as an icebreaker as participants did not know each other yet. Its purpose was to make participants feel at ease and create a safe atmosphere to share experiences and opinions [33]. Participants were asked to choose a photo from a variety of options that they felt best captured their experiences with working with the EHR. Because participants already knew each other in the other four focus groups, the photo elicitation method was deemed less relevant and, therefore, not used.

A semi-structured interview guide was created and tailored to each focus group, reflecting each discipline. Moreover, adaptations were made based on insights from previous focus groups [34].

All participants were asked to discuss two main topics: the use of the EHR in daily care for older people, including what care professionals need to work with the EHR and what they want to read in the EHR, and the use of EHRs for quality improvement. At the end of each focus group, participants were encouraged to raise any additional relevant topics. Data collection continued until data saturation was reached.

### Data analysis

Conventional content analysis was used to analyze the focus groups. This allows themes to arise without being guided by preconceived theory [35]. First, the audio recordings of the focus groups were transcribed verbatim using internal software developed at Maastricht University (STT model) [36]. These transcripts were manually checked and then uploaded to the qualitative data-analysis software MAXQDA24 [37]. Second, RV read the transcripts to become familiar with the content, and then coded two focus groups and discussed these with a research assistant. Third, RV coded all focus groups and identified preliminary themes. Hereafter, to validate the code tree, the research assistant coded two focus group transcripts with the existing codes. Discussions between them led to the resolution of discrepancies in coding. Afterwards, a group of sparring partners (*n* = 3), consisting of a registered nurse, certified nurse assistant, and elderly care physician, convened for a face-to-face meeting in which they were presented with the preliminary results. Their input served as additional validation of these results, which were further nuanced, yet not changed content-wise. Finally, a discussion between KS, YG, and RV led to further refinement of the themes.

### Trustworthiness

Several approaches were used to enhance the trustworthiness of this study. First, triangulation was used in two ways. Data triangulation was employed by recruiting participants with various professional roles for the focus groups. Investigator triangulation was used by moderating the focus groups in pairs, drawn from a pool of four researchers. All have experience in qualitative interviewing to a varying extent. Data-analysis was performed by one of the focus group moderators and a research assistant who did not moderate the focus groups, thereby providing a neutral and critical perspective in analyzing the data. Regular discussions within the research team facilitated critical reflection on emerging interpretations [38]. Second, many individuals participated in the focus groups, creating a rich dataset. Emerging themes demonstrated overlap across focus groups, indicating that data saturation was reached [39]. This was confirmed by representatives of each profession in the sparring partners meeting. Third, participants were encouraged to express their views and were invited to add any relevant information they felt was not covered in our questions, to ensure that the findings reflect the true opinions of the participants. Finally, transferability of the results was increased by supporting the reported themes with participant quotes [40].

### Ethical considerations

This study received ethical approval from the medical ethics committee of Zuyderland (METCZ20230066). Participants were informed in advance about the aims and voluntary nature of the study. Before each focus group, participants were informed about the study’s purpose and procedures. Written informed consent (or verbal in case of the online focus group) was provided by all participants. Privacy of the participants was ensured by pseudonymizing all participant data during transcription of the audio-recordings. Anonymous data is stored on a secure server of Maastricht University.

## Results

A total of 18 care professionals and 25 student care professionals attended six focus groups between November 2023 and February 2024. After the sixth focus group, data saturation was reached. Focus groups lasted between 26 and 122 min. Table 1 presents an overview of the participants in each focus group.

**Table 1:** Focus group participants

Focus group	Discipline/profession	Total (*N* = 43)
1	Registered nurses and certified nurse assistants	6 (14%)
2	Registered nurses and certified nurse assistants	4 (9%)
3*	Elderly care physicians	8 (19%)
4	Student certified nurse assistants	15 (35%)
5	Student registered nurses	4 (9%)
6	Student elderly care physicians	6 (14%)

*N* Number

*conducted online

Analyses revealed three main themes: (1) different purposes of the EHR (2), navigating the technical aspects of EHR systems in daily use, and (3) supporting interprofessional collaboration through the EHR. Each main theme can be divided into subthemes (Table 2).

**Table 2:** Themes describing participants’ experiences of working with the EHR

Themes	Subthemes
Different purposes of the EHR	The EHR as communication tool: balancing different stakeholder needs
	A foundation for data-informed care
Navigating the technical aspects of EHR systems in daily use	Skill development in EHR use
	Diversity and technical challenges in EHR systems
Supporting interprofessional collaboration through the EHR	Different opinions on what “good reporting” means
	Towards improved collaboration with the EHR for high-quality care

### Different purposes of the EHR

The first identified theme was: different purposes of the EHR. This theme encompasses various purposes that participants envisioned for the EHR. These purposes could be differentiated between objectives for which the EHR is already used, and objectives that the EHR could potentially fulfil in the future. The two subthemes reflect this differentiation, as they represent a current and a future-oriented purpose, respectively: (a) the EHR as a communication tool: balancing different stakeholder needs, and (b) a foundation for data-informed care.

#### a. The EHR as a communication tool: balancing different stakeholder needs

As a first purpose, participants in all focus groups indicated that the EHR serves as a communication tool for informing colleagues, residents, residents’ relatives, and third parties, such as case managers. It both stores information, such as residents’ histories, preferences, routines, and care agreements, and acts as an up-to-date log of daily care activities, medication changes, and residents’ well-being.

*“The continuity [in staff] is difficult to ensure at the moment*,* so you have weekly resident visits with someone else […]. So I use the EHR to verify: what did actually happen?”* – focus group 6, student elderly care physician

*“If I have a morning shift*,* I read the reports and the care plan the evening before. Then I see for example that a colleague reported: this needs to happen*,* this still needs to happen. And then I think: o good*,* because then I know what is awaiting me in the morning.”* – focus group 4, student certified nursing assistant

A few times across the various groups, participants highlighted that with this reported information, the EHR serves as support for delivering high-quality care:

*“Based on what we report and the way of questioning in the EHR*,* you give a certain direction for the good care that you want to deliver.”* – focus group 2, registered nurse/certified nurse assistant

However, it was also acknowledged that delivering high-quality care was not experienced by all care professionals as the main goal of the EHR:

*“Some people fill it [the EHR] in on autopilot [without reflection]*,* and then we’re done. But why you do that is no longer known.”* – focus group 2, registered nurse/certified nurse assistant

*“It [the EHR] should be helping. But I notice that it depends a lot on the ward and the quality of reports. Sometimes a picture is thrown in the EHR of a body part*,* no clue what*,* and then @physician: do something with this. In my opinion it is abused to move the problem elsewhere.”* – focus group 6, student elderly care physician

Participants, especially the student registered nurses, recognized that the EHR’s information-sharing enables two-way communication with families. That is, in the Netherlands, residents and their families have access to the EHR and can therefore read along with the reports of care professionals. In this context, student registered nurses acknowledged that families play an important role in sharing details about the residents’ background and preferences, while care professionals inform families about the residents’ daily lives in the nursing home. Awareness was shown of the information function the EHR serves for relatives. They indicated they sometimes write reports specifically for relatives, as illustrated by the following quote:

*“And family members also appreciate it more*,* because it is about well-being*,* like*,* also write something nice for that family at times and not just: ‘had to correct Mrs. because she was hitting the neighbor’.”* – focus group 1, registered nurse/certified nurse assistant

Yet, some participants, specifically elderly care physicians in training, expressed concerns about EHR accessibility for relatives. They noted that the language used in EHR is intended for colleagues, not relatives, and may therefore be perceived as blunt or insensitive. Moreover, they experienced that the immediate availability of information for relatives sometimes led to unwanted situations, and thereby questioned its desirability, as illustrated by the following comment:

*“We had that once with a suicide attempt. The physician had already written everything down*,* and the nurses as well*,* but the family had not been informed; we hadn’t been able to reach them*,* and they read it through the record. So that was really terrible.”* – focus group 6, student elderly care physician

#### b. A foundation for data-informed care

A focal point among elderly care physicians was the potential for EHR data to support data-informed care in nursing homes. While this topic was frequently discussed among this group, it received little to no attention from registered nurses, certified nurse assistants, or the three groups of student care professionals.

When discussing data-informed care, the elderly care physicians suggested that EHR data could be used not only for individual care but also at the ward and organizational level to enhance the quality of care. For example, they said data could help tailor interventions to address specific ward needs, such as reducing falls or medication errors.

*“If more medication errors are made in one ward*,* or there are more falls*,* then you would like to address this quickly*,* and of course*,* we are already doing that*,* but we would like to continuously do better.”* – focus group 3, elderly care physician

The discussions among physicians about data-informed care were future-oriented, recognizing that their ideal of data-informed care does not reflect how the EHR is currently used. Elderly care physicians wish for a broader application of EHR data beyond daily care provision. For example, they pose that data can be used as a starting point for learning and quality improvement.

*“What we also want to do […] is look at the [staff] formation […]. We need to move in two directions*,* so deploying the capacity that we have very well*,* but also predicting where problems will arise and preferably getting ahead of them.”* – focus group 3, elderly care physician

### Navigating the technical aspects of EHR systems in daily use

The second identified theme was navigating the technical aspects of EHR systems in daily use. Participants described challenges of working with the EHR system in their daily work, both on a personal level and regarding the EHR system. Two subthemes were identified: (a) skill development in EHR use and (b) diversity and technical challenges with the EHR system.

#### a. Skill development in EHR use

Competencies for working with the EHR were discussed by registered nurses and certified nurse assistants, and to some extent, student elderly care physicians. It was felt that proficiency with the EHR is not an automatic skill. Clinical reasoning, reporting, and computer skills were mentioned most frequently as necessary to navigate daily work with the EHR. The following quotes illustrate that participants were not always satisfied with their colleagues’ reporting proficiency:

*“At times I wonder where the line is to be allowed to report in the electronic health record*,* because I sometimes worry when you see the quality of reports you already encounter now.”* – focus group 6, student elderly care physician

*“Now I sometimes see improvement*,* but it tends to be that a lot of people just find the computer very scary*,* and don’t know what to do with it.”* – focus group 1, registered nurse/certified nurse assistant

Participants commonly indicated encountering reports that were overly long and unstructured. They cited examples of colleagues who were not proficient in Dutch and lacked the writing skills to effectively communicate through written text. The following quotes illustrate this:

*“Sometimes I am searching and reading things*,* and then I think: yes*,* it first says ‘care according to plan’ 10*,*000 times. And then I am scrolling and think: it takes up so much of my time*,* while I need to get to the heart of it.”* – focus group 5, student registered nurse

*“Everything is reported under one heading*,* and some colleagues just make it a wall of text*,* without spaces*,* no full stops*,* no commas*,* nothing.”* – focus group 4, student certified nurse assistant

All but the elderly care physicians’ focus group discussed how care professionals learn how to work with the EHR. Although they indicated they often learn reporting skills during their education, they experienced that workplace practices strongly influence how reporting is done. Students noted that they were expected to adopt their employer’s specific reporting method and that learning how to report was largely guided by training from the care organization and colleague support. Still, in spite of the existing training offer to learn to work with the EHR, not all colleagues appear open to learn.

*“We have had*,* at least for half a year*,* I don’t know how many meetings*,* presentations*,* to grow awareness of the SOAP [reporting method] notes.” –* focus group 2, registered nurse/certified nurse assistant

*“But that brings us back to the digital side of things. Not everyone has the digital skills or knows how to use it […]. And some find it easy to leave it to others and say: ‘You do the reporting*,* then I’m done with it.’ Yes*,* you have those people too.” –* focus group 2, registered nurse/certified nurse assistant

#### b. Diversity and technical challenges with the EHR system

Participants in all focus groups expressed concerns about the EHR system. A commonly mentioned issue was the use of various operating systems. These different systems often fail to connect, which hinders the transfer of client data to another organization, for example, when moving from home care to a nursing home.

*“Why do there have to be so many different types of EHRs or so many different systems that you can work with? Just make one good one*.” – focus group 6, student elderly care physician.

Difficulties are not only encountered between organizations, but also between disciplines. For example, the medical records for elderly care physicians were often not integrated within the EHR. As a result, information may not be transferred automatically, making it difficult to locate essential data. In turn, collaboration between disciplines is affected by this set-up with two types of records.

*“What I notice mostly is that in the EHR things are suggested [by nursing staff]. ‘Maybe we should culture a urine sample […].’ The family can already read this and expect something from you as a physician. But the family cannot read the medical reports. […] So you already feel indirectly pressured to set in motion certain actions.” –* focus group 6, student elderly care physician.

Moreover, participants indicated that even systems from the same manufacturer may look different, as organizations can either choose to buy the basic settings or purchase access to additional features. This also makes it more complicated for care professionals to learn to work with the EHR.

*“It depends on what functions you have [in the EHR] in your organization. You have to buy packages. And for example*,* I cannot categorize advance care planning somewhere. There is a nice header for that [in the system]*,* but it is not available to us. So*,* that is not useful.”* – focus group 5, student registered nurses.

### Supporting interprofessional collaboration through the EHR

The third theme was: supporting interprofessional collaboration through the EHR. Participants talked about their wish to be able to effectively collaborate with peers and professionals across disciplines. Participants noted that the EHR’s potential as a supportive tool in such interprofessional collaboration is not yet fully reached. Two subthemes were distinguished: (a) different opinions on what "good reporting" means, and (b) towards improved collaboration with the EHR for high-quality care.

#### a. Different opinions on what “good reporting” means

Inconsistency in reporting was a common topic of discussion among all participants, except the group of elderly care physicians. The opinions of participants on what they consider to be good reporting differed and could not be separated along professional lines. The first disagreement was regarding the amount of information that is to be reported. One group of participants expressed only wanting to read the bare minimum of text in the EHR, for example, sudden changes or special details. The other group of participants voiced the opinion that there is always something to report, which is illustrated by the comment below:

*“If you went to a client*,* I don’t think you can say there is never anything [to report] […] You are always doing something. The care can go well*,* but I actually think you always have something to report. Yes. It seems strange that you go to a client and say*,* there was nothing.”* – focus group 2, registered nurse/certified nurse assistant.

A second point of disagreement concerned reporting from a health versus well-being perspective. This debate was mainly waged among registered nurses and certified nurse assistants. According to them, some disciplines were more inclined towards reporting on well-being, such as activity coordinators and well-being employees. These divergent views on reporting priorities may lead to frustration with colleagues on reporting styles:

*“For example*,* I think it is important to report on someone’s behavior and mental health. And others*,* in turn*,* think*,* it [reporting] is only on physical health. […] That is sometimes very difficult.”* – focus group 2, registered nurse/certified nurse assistant.

Nevertheless, some participants disagreed with the idea that health and well-being are equally important, and asserted that health should be seen as a prerequisite for well-being:

*“If someone really does not feel well*,* has a low blood sugar*,* then I can do what I want*,* but he will continue to feel incredibly bad*,* then I first have to do something about that. That is then health-related*,* and after I can return to well-being.”* – focus group 1, registered nurse/certified nurse assistant.

Moreover, participants mentioned that reporting style is partly governed by personal preferences, but indicated that organizational demands also steer the reporting style of care professionals.

*“Yes*,* every organization has its own way of reporting. One reports very short*,* while another writes stories. At my organization*,* we do not report if it is not necessary*,* so I go along with that.”* – focus group 4, student certified nurse assistant.

#### b. Towards improved collaboration with the EHR for high-quality care

Elderly care physicians, registered nurses, and certified nurse assistants expressed a desire for good collaboration between care professionals to achieve high-quality care for residents. The EHR was identified as a useful tool to attain this goal.

*“You have all those disciplines and people who work in the EHR to make it one*,* in order to be able to deliver quality and good care.”* – focus group 2, registered nurse/certified nurse assistant.

The student elderly care physicians, student certified nurse assistants, and the two groups of registered nurses and certified nurse assistants mentioned which stakeholders should be part of such a team. Multiple times, it was stressed that residents and family should be involved in the decision-making processes and when making agreements on the care plan. Other stakeholders were also identified.

*“As far as I’m concerned*,* multidisciplinary means the full range. So not just the physician and care professionals and the psychologist*,* […] but all allied health professionals you possibly employ*,* but also management*,* sometimes facility services*,* and certainly family could possibly play a role towards the future. So*,* for me*,* multidisciplinary really means the full range.”* – focus group 3, elderly care physician.

Yet, to reach this desired interprofessional collaboration, several barriers need to be overcome first, according to participants in all focus groups. For example, care professionals need to realize the value of collaboration, and all disciplines need to be present during interprofessional team meetings. And although good quality reporting in the EHR is considered important, participants indicated that it does not replace face-to-face communication.

*“And then the elderly care physician rightfully says*,* this is not a multidisciplinary team meeting if I only sit here with family and the staff [without allied health professionals]*,* that is just a conversation*,* that is not really a multidisciplinary team meeting.”* – focus group 2, registered nurse/certified nurse assistant.

*“No*,* I also don’t think reporting is a replacement for the verbal briefing. The verbal briefing between night shift and morning shift*,* but also the communication between the first contact person and the family.”* – focus group 6, student elderly care physician.

Another challenge regarding interprofessional collaboration with the EHR is the lack of clarity about the roles and responsibilities of care professionals. Participants regularly cited poor follow-up of tasks and agreements that are documented in the EHR as a result of this unclarity. For instance, when the condition of a resident deteriorates and a treatment has been started, participants reported that feedback on the results of the treatment were often missing in the EHR, and that it was not clear who was responsible for this follow-up, leading to suboptimal care for the resident.

*“If it concerns the monitoring of someone who is not well or where you diagnosed an infection […] and you ask colleagues to do check-ups and make observations. It is the human factor that is the weakest link there*,* so you are already very happy if you agree on [reporting] two times a day*,* that it is reported once every three days in a manner of speaking.”* – focus group 3, elderly care physician.

A final challenge in interprofessional collaboration with the EHR concerned a perceived hierarchy, with certified nurse assistants and registered nurses reporting the feeling that their EHR reports had to meet the standards of elderly care physicians.

*“We also noticed in discussions that the question arose: who are we actually reporting for? […] You saw with the elderly care physician that it was like*,* ‘I am here*,* everything passes through me*,* so to speak. I need to be involved in every decision that is made.’ So you end up reporting accordingly […]. And in that sense*,* you are actually reporting for the physician and not for the client.”* – focus group 2, registered nurse/certified nurse assistant.

## Discussion

This qualitative study investigated how different disciplines of (student) care professionals in nursing homes view working with EHRs. The analysis revealed three themes in which both similar and different views were identified between the participants, being the certified nurse assistants, registered nurses, and elderly care physicians. First, the EHR is mainly used as a communication tool, and further purposes, such as data-informed care, were pointed out as goals to be realized in the future. Second, better navigation of the EHR system necessitates skill development among EHR users and improvements in the technical functions of the EHR system. Finally, the EHR can support interprofessional collaboration in the care for older people. Yet, care professionals recognized that there is still room for improvement in such a collaboration.

The findings show that care professionals conceptualize the goal of the EHR largely in the same way, namely, as a communication tool to keep stakeholders informed about the daily care of residents and to improve the quality of care. This aligns with other findings indicating that the EHR can have a positive impact on quality indicators and quality of care [41, 42]. Nonetheless, improvements to the EHR remain, such as aligning the operating systems of different EHRs. Improving the usability of EHR systems for care professionals has previously been reported as a challenge in long-term care (LTC) [14], demonstrating that there is no straightforward approach to address these challenges. As EHR user preferences have been shown to influence the success of EHR implementation in LTC organizations [43], it is important to continuously invest in improving the EHR system to fit these preferences.

Elderly care physicians specifically emphasized the importance of data-informed care in the care for older people. According to them, the wealth of data in EHRs should be used more extensively for quality improvement in LTC, in line with previous research [44]. This stance corresponds with the shift towards data-informed care [45], yet simultaneously shows we are still at the start of this movement. Elderly care physicians identified several obstacles to data-informed care. Echoing previous studies, a frequently mentioned barrier was a lack of uniformity in reporting [16]. Easy reuse of data requires structured and systematic documentation, even though such a one-size-fits-all format might not be suitable for the complex reality of everyday use [46]. While data-informed care was a common theme among elderly care physicians, other groups merely discussed quality improvement on a case-by-case basis. This may reflect the semi-structured nature of the focus groups, in which moderators allowed participants to steer the discussion towards issues they considered most relevant rather than systematically covering all topics in equal depth. At the same time, the limited attention given to quality improvement may in itself be meaningful. It could indicate that participants perceived this topic as laying outside the scope of their daily practice and as less salient than other issues emerging during the discussions.

Besides the challenges associated with the uniformity of reporting, another significant issue is the inconsistency in the design of different EHR systems. In 2025, 101 care organizations caring for older adults in the Netherlands used EHRs from nine suppliers [47]. It is debatable whether we can expect care professionals to be able to effectively work within this complex landscape. Yet, having these technological skills is included in the list of professional competencies required of certified nurse assistants, registered nurses, and elderly care physicians in the Netherlands. Care professionals should possess seven main competencies outlined in the CanMEDS framework, which describes competencies of care professionals in several countries, including the Netherlands [48]. Communicator and manager are two of these competencies. As part of these competencies, care professionals are expected to effectively communicate with others, including the proper use of IT [49, 50]. However, our findings indicated that care professionals’ digital literacy is not currently sufficient to meet the CanMEDS requirements. Therefore, we should be aware of the necessity of supporting care professionals in developing digital skills [16].

Another finding revealed the potential of the EHR to support care professionals in fostering interprofessional collaboration. However, when zooming in on how interprofessional collaboration looks in practice, its implementation requires further consideration. Interprofessional team collaboration can be conceptualized by eight domains [51], and not all domains seem to be fully represented in the described collaboration. For example, learning capacity is a dimension that, according to participant descriptions, does not tend to reach its full potential. Participants described situations where their colleagues lacked computer and writing skills, and learning was identified as a way to gain these skills. However, a proactive attitude towards learning seemed to be lacking at times, despite the fact that urgency to learn is a precondition for workplace learning [52]. For example, some care professionals reportedly asked their colleagues to report for them in the EHR, instead of investing time in improving their own skills. Another dimension of interprofessional team collaboration in which participants identified room for improvement was group dynamics and interaction [51]. An illustration of this is the sentiment of certified nurse assistants and registered nurses that the elderly care physician is in charge and does not always value their input. This negative experience resonates with findings showing that hierarchy can lead to lower team effectiveness. Hierarchy can especially have a negative impact when team members have a very diverse skill set, which is the case in interprofessional teams in nursing homes [53]. Thus, although care for older people is led by interprofessional collaboration, the EHR is not yet being used to its full potential in this collaboration.

This is one of the first studies to research the use of the EHR from different perspectives, including students. A strength of this study is the inclusion of both care professionals and student care professionals as participants, the latter representing the workforce of the future. While care professionals have more work experience, students have first-hand knowledge of their program curricula, allowing them to use the most recent expertise in practice. Interestingly, apart from their differing perspectives on learning, the two groups do not differ much in stances or discussion topics. When students discussed learning, they talked about learning in the context of their educational program. In contrast, experienced care professionals pointed out that the care organization is responsible for providing learning opportunities. The latter group also discusses prerequisites for workplace learning, such as repetition and willingness to learn, which students did not discuss. On the one hand, this distinction is unsurprising, as participants naturally emphasized the learning context most relevant to their role. On the other hand, the similarity in perspectives suggests that students may already be aligning with workplace norms, reflecting early professional socialization through their internships in practice.

Nevertheless, there are some methodological considerations that should be noted. First, due to recruitment challenges, it was not feasible to conduct separate focus groups for certified nurse assistants and registered nurses as originally planned. Instead, participants from both professional groups were included in two separate focus groups in different regions. The mixed composition of the focus groups may have reduced participants’ willingness to openly discuss profession-specific perspectives, which may have limited the exploration of differing viewpoints. However, we chose to proceed with the mixed focus groups in order to ensure participation from both professional groups, rather than excluding them due to recruitment constraints. Second, other professions in the care for older people, such as physical therapists, well-being professionals, and dentists, were not included as participants, despite being part of the nursing home teams and therefore a part of interprofessional learning and quality improvement efforts. Because the team of caregivers providing basic care consists of certified nurse assistants, registered nurses, and elderly care physicians, who all work with the EHR frequently, it was chosen to only include these professions. Third, the photo elicitation method was only used in the two focus groups composed of previously unacquainted participants, while the remaining groups directly started the discussion. Whereas it must be acknowledged that opening the focus groups differently may have influenced group dynamics, all groups engaged in lively discussions. A final methodological consideration is that the homogeneous nature of the focus groups did not reflect the interprofessional character of care professionals’ work. The choice was made for this set-up because it allowed distilling shared beliefs within professions. Future studies on interprofessional collaboration in nursing home care could, therefore, focus on bringing these perspectives together in heterogeneous focus groups.

It is recommended that future studies adopt a wider understanding of the interprofessional team to include more professions. Similarly, clients, client representatives, and family members should be included. This recommendation aligns with the shift towards relationship-centered care [54, 55], while also fostering a greater feeling of ownership over one’s own data in the EHR when these groups are included in the interprofessional team. Moreover, it is recommended that uniformity in EHR reporting be focused on increasing mutual understanding and follow-up. The input from both users and IT specialists should be included to incorporate user preferences to the largest extent possible within the EHRs’ technical boundaries. Furthermore, future studies should target the competencies of care professionals required to report in the EHR to ensure reporting of sufficient quality. These studies should integrate learning during the educational program and the role of workplace learning in continuously updating the necessary competencies.

## Conclusions

This study shows the views of different disciplines of (student) care professionals in nursing homes on working with the EHR. Although some nuances can be observed between professional groups, their views are generally consistent. For the EHR to be effectively used as a communication tool, uniformity in EHR systems is desired, and greater digital literacy is expected from care professionals. Moreover, care organizations should be aware that a group of employees working together in an EHR does not necessarily function as an interprofessional team. For a team to thrive, time should be spent on creating the necessary conditions for interprofessional collaboration. Future studies should investigate what interprofessional teams need to make better use of data to improve quality of care in the care for older adults in nursing homes.

## Data Availability

The data that were analyzed during the current study are available from the corresponding author upon reasonable request.

## Appendix

Example questions from the interviews:

- What do you write down in the EHR and why?
- What information do you want to read in the EHR?
- How does the EHR help you in your work as [position]?
- What can be improved in the EHR?
- How is the transfer from shift to shift? Do you use the EHR for this and how?
- How do you approach multidisciplinary working?
- What do you learn during your education about the EHR?
- How does your education help you in working with the EHR?
- What do you need to use the information in the EHR to do your job as good as possible?
- Do you ever use the information in the EHR to improve the care for individual clients? Do you ever use this to improve the quality of care on the ward level?
- What do you need to be able to work effectively with the EHR?
- How should the EHR look in the ideal world?

## Abbreviations

EHR: Electronic Health Record
STT-model: Speech-To-Text model
LTC: Long-term care
